# Mesh vs. non-mesh repair of type I hiatal hernias: a propensity-score matching analysis of 6533 patients from the Herniamed registry

**DOI:** 10.1007/s10029-024-03013-z

**Published:** 2024-03-29

**Authors:** H. Hoffmann, P. Glauser, D. Adolf, P. Kirchhoff, F. Köckerling

**Affiliations:** 1 ZweiChirurgen GmbH, Center for Hernia Surgery and Proctology, Basel, Switzerland; 2 Merian Iselin, Clinic for Orthopedics and Surgery, Basel, Switzerland; 3 Solothurn Hospitals, Clinic for Surgery, Dornach, Switzerland; 4 StatConsult GmbH, Magdeburg, Germany; 5 Hernia Center, Vivantes Humboldt Hospital, Academic Teaching Hospital of Charitè University Medicine, Am Nordgraben 2, 13509 Berlin, Germany

**Keywords:** Hernia repair, Hiatal hernia, Reflux, GERD, Mesh, Herniamed

## Abstract

**Introduction:**

Surgical treatment of type I hiatal sliding hernias aims to control the gastroesophageal reflux symptoms and prevention of hernia recurrence. Usually, a cruroplasty is performed to narrow the hiatal orifice. Here, it remains controversial if a mesh reinforcement of the cruroplasty should be performed, since benefits as well as mesh-associated complications have been described.

**Methods:**

We performed a propensity-score matching analysis with data derived from the Herniamed registry comparing patients undergoing laparoscopic type I hiatal hernia repair with and without synthetic mesh. We analyzed perioperative, intraoperative, and postoperative data including data derived from the 1-year follow-up in the registry.

**Results:**

6.533 patients with an axial, type I hiatal hernia and gastroesophageal reflux are included in this analysis. Mesh augmentation of the hiatoplasty was performed in *n* = 1.252/6.533 (19.2%) of patients. The defect size in the subgroup of patients with mesh augmentation was with mean 16.3 cm^2^ [14.5; 18.2] significantly larger as in the subgroups without mesh augmentation with 10.8 cm^2^ [8.7; 12.9]; (*p* < 0.001). In patients with mesh hiatoplasty *n* = 479 (38.3%) Nissen and *n* = 773 (61.7%) Toupet fundoplications are performed. 1.207 matched pairs could be analyzed. The mean defect size after matching was with 15.9 cm^2^ comparable in both groups. A significant association was seen regarding recurrence (4.72% mesh vs. 7.29% non-mesh hiatoplasty, *p* = 0.012). The same relation can be seen for pain on exertion (8.78% vs 12.10%; *p* = 0.014) and pain requiring treatment (6.13% vs 9.11%; *p* = 0.010). All other outcome parameter showed no significant correlation.

**Conclusions:**

Our data demonstrate that mesh-reinforced laparoscopic type I hiatal hernia repair in larger defects is associated with significantly lower rates for recurrence, pain on exertion and pain requiring treatment.

## Introduction

Hiatal hernia describes a dilated esophageal hiatus through which the gastroesophageal junction protrudes, and in the maximum variant the entire stomach and other intestinal organs can be displaced in the thorax [1]. There are four types of hiatal hernias (I–IV), with type I (sliding hernia) being the most frequent variant. Intra-abdominal pressure (e. g. chronic obstructive pulmonary disease or chronic constipation) and congenital causes are assumed to be the causes of hiatal hernias [2]. Around 80–90% of hiatal hernias are associated with gastroesophageal reflux disease (GERD), especially in type I hernias [3]. The indication for surgical treatment depends on the extent of the hernia morphology and the clinical symptoms.

Surgical treatment in type I hiatal hernias (sliding hernias) predominantly aims to control the GERD symptoms if medical treatment fails [3–5]. Here, usually anti-reflux techniques such as a Nissen or Toupet fundoplications are used, creating a sleeve around the gastroesophageal junction to reinforce the distal esophageal sphincter [1]. In most procedures a cruroplasty is performed to narrow the hiatal orifice [6]. Although recurrence rates of 59% following pure suture cruroplasty after 5 years are described [7] it still remains controversial, if mesh reinforcement of the cruroplasty should be performed [8]. Guidelines from the Society of American Gastrointestinal and Endoscopic Surgeons (SAGES) and the European Association of Endoscopic Surgery (EAES) see advantages in terms of a lower recurrence rate after mesh reinforcement, but do not recommend routine mesh implantation in the treatment of GERD of type I hiatal hernias due to insufficient literature evidence and a relevant mesh-associated complication potential such as dysphagia and erosion of the esophagus [5]. As a consequence, indications for mesh should be limited to patients with weak crurae and a larger hiatal defect [9]. The benefit of using a mesh augmentation of the cruroplasty in type I hernias may not outweigh the concerns of mesh-associated complications. However, there are some data supporting the use of a mesh in type I hiatal hernias augmenting the cruroplasty [9–11] reducing recurrence and intrathoracic wrap herniation. For decision pro or against a mesh augmentation, some authors suggest a patient tailored algorithm, taking hiatal hernia type, hiatal diameter, tropism of the hiatal pillars and presence of recurrence into account [7–13] But the controversy underlines the lack of high-evident data to support or refuse a routine mesh reinforcement of the cruroplasty during type I hiatal hernia repair.

Therefore, this matched pair analysis investigates the effect of mesh augmentation of the cruroplasty during type I hiatal hernia repair with data derived from the Herniamed registry.

## Methods

The Herniamed Registry is a multicenter, internet-based hernia registry [12] with 936 participating hospitals and surgeons (status April 2023) in private practice (Herniamed Study Group) in Germany, Austria, and Switzerland who have entered data on their patients undergoing routine hernia surgery. All patients signed an informed consent agreeing to participate. As part of the information provided to patients regarding participation in the Herniamed Quality Assurance Study and signing the informed consent declaration all patients were informed that the treating hospital or medical practice should be informed about any problem occurring after the operation and that the patient should have a clinical examination if needed. All postoperative complications occurring up to 30 days after surgery were recorded. At 1-year follow-up, postoperative complications were once again reviewed when the general practitioner and patient completed a questionnaire [12]. At 1-year follow-up, the general practitioner and the patient were also asked about any recurrences, pain at rest, pain on exertion, and chronic pain requiring treatment. If a recurrence, pain, secondary hemorrhage, or blood effusion are reported by the general practitioner or the patient, the patient can be requested to attend clinical examination.

The present retrospective analysis of prospective data compares the perioperative and 1-year follow-up data between October 2009 and March 2022 in patients undergoing type I hiatal hernia repair. Inclusion criteria were fully documented elective primary laparoscopic axial hiatal hernia repairs with Toupet or Nissen fundoplication in patients with GERD, minimum valid age of 16 years and complete 1-year follow-up. In a registry the individual surgeon of the participating institutions decide about the type of fundoplication and the additionally use of a mesh.

All statistical analyses were performed using the software SAS 9.4 (SAS Institute Inc., Cary, NC, USA) and intentionally calculated to a full significance level of 5%, i.e., they were not corrected with respect to multiple testing. For unadjusted comparison of parameters between female and male patients (to present the differences between the original pre-matched samples), *χ*2 test and *t* tests (Satterthwaite) were performed for categorical and continuous variables, respectively.

Pairs of mesh and non-mesh patients were matched using a 1:1 propensity-score matching with a greedy algorithm and a caliper of 0.2 standard deviations. The variables used for matching were as follows: age, weight and morbid obesity (classification of body mass index (BMI, kg/m^2^)), defect size, American Society of Anesthesiologists (ASA) status, preoperative pain, regurgitation, dysphagia, anemia/hemorrhage, lung disorders and presence of at least one further risk factor (chronic obstructive pulmonary disease, diabetes mellitus, aortic aneurysm, immune suppression, cortisone medication, smoking, coagulopathy, anticoagulant, or antiplatelet medication) as well as sex and type of fundoplication (Nissen, Toupet). The last two were used as fixed matching variables, i.e., there must be no deviation within the matched pair.

The balance of the matched sample was assessed using standardized differences (also given for the pre-matched sample), which should not exceed 10% (< 0.1) after creating matched pairs. Matched samples were then analyzed for intraoperative complications, general complications, postoperative complications, complication-related reoperations, recurrence on 1-year follow-up, pain on exertion on 1-year follow-up, pain at rest on 1-year follow-up, pain requiring treatment on 1-year follow-up, trocar hernia on 1-year follow-up, secondary hemorrhage on 1-year follow-up, blood effusion on 1-year follow-up and infection on 1-year follow-up via McNemar’s test. The results obtained are presented as the concordant and discordant results per matched pair within a 2 × 2 frequency table. Further, the corresponding p values and the odds ratio (OR) estimates for matched samples with 95% confidence interval are included in a forest plot of the results.

## Results

### Univariate analysis

**Fig. 1:**
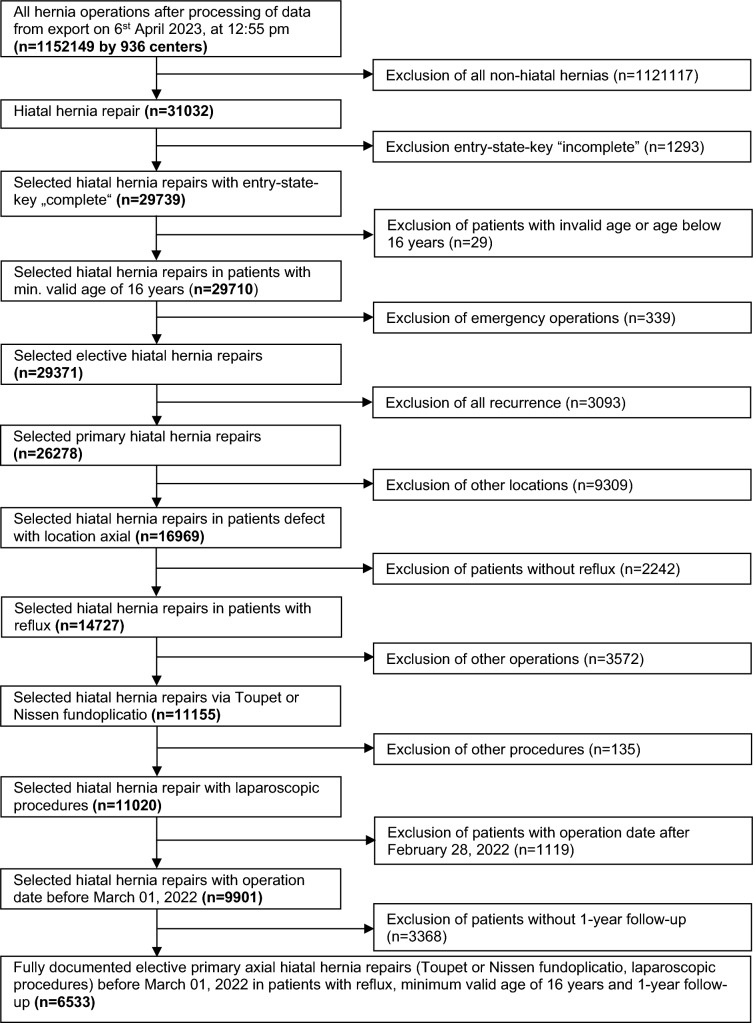
Flowchart of patient inclusion

In total 6533 patients were included in the analysis (Fig. 1), mesh patients composed 19.16% (*n* = 1252) of the population. The defect sizes in the subgroup of patients with mesh augmentation was with mean 16.3 cm^2^ [14.54; 18.2] versus 10.8 cm^2^ [8.7; 12.9] significantly larger compared with the subgroub witout the use of mesh (*p* < 0.001) (Table 1). Unadjusted comparison of the patient and operation characteristics before matching (Table 2) showed significant differences between mesh vs. non-mesh repair for the preoperative pain, surgical technique, regurgitation, dysphagia, and pain (*p* < 0.001, each). Table 3 presents the unadjusted analysis of postoperative outcome before matching. After 1 year of follow-up mesh vs. non-mesh patients showed no significant difference in all outcome parameter except pain on exertion which tends to occur more frequently in non-mesh repair (*p* = 0.072).

**Table 1:** Presentation of ranges and of unadjusted analysis results for homogeneity between mesh and non-mesh hiatoplasty for age and defect size

		Mesh		
		Yes	No	*p*
Age [years]	*N*/mean ± SD	1252/57.9 ± 13.3	5281/56.0 ± 13.7	< 0.001
Defect size [cm^2^]^a^	*N*/mean [range of dispersion]	1252/16.3 [14.5; 18.2]	5281/10.8 [8.7; 12.9]	< 0.001

^a^ Logarithmic transformation: presentation of the back-transformed mean values and ranges (mean value ± SD)

**Table 2:** Unadjusted comparison of mesh versus non-mesh hiatoplasty for patient and operation characteristics before matching

		Mesh				
		Yes		No		
		*n*	%	*n*	%	*p*
Sex	Male	508	40.6	2280	43.2	0.095
	Female	744	59.4	3001	56.8	
BMI	Underweight	7	0.6	31	0.6	0.742
	Normal weight	315	25.3	1266	24.1	
	Overweight	557	44.8	2437	46.4	
	Obesity/morbid	365	29.3	1522	29.0	
ASA	I	235	18.8	1032	19.5	0.309
	II	842	67.3	3437	65.1	
	III/IV	175	14.0	812	15.4	
Preoperative pain	No	328	26.2	2083	39.4	< .001
	Yes	810	64.7	2675	50.7	
	Unknown	114	9.1	523	9.9	
Operation technique	Nissen fundoplication (360°)	479	38.3	2560	48.5	< .001
	Toupet fundoplication (270°)	773	61.7	2721	51.5	
Regurgitation	Yes	431	34.4	1098	20.8	< .001
	No	821	65.6	4183	79.2	
Dysphagia	Yes	301	24.0	978	18.5	< .001
	No	951	76.0	4303	81.5	
Pain	Yes	551	44.0	1683	31.9	< .001
	No	701	56.0	3598	68.1	
Anemia/hemorrhage	Yes	46	3.7	151	2.9	0.130
	No	1206	96.3	5130	97.1	
Lung disorders	Yes	86	6.9	328	6.2	0.390
	No	1166	93.1	4953	93.8	
Risk factors—total	Yes	276	22.0	1318	25.0	0.031
	No	976	78.0	3963	75.0	
COPD	Yes	101	8.1	463	8.8	0.428
	No	1151	91.9	4818	91.2	
Diabetes	Yes	55	4.4	212	4.0	0.543
	No	1197	95.6	5069	96.0	
Aortic aneurysm	Yes	0	0	10	0.2	0.123
	No	1252	100	5271	99.8	
Immunosuppression	Yes	6	0.5	39	0.7	0.319
	No	1246	99.5	5242	99.3	
Corticoids	Yes	20	1.6	60	1.1	0.182
	No	1232	98.4	5221	98.9	
Smoking	Yes	88	7.0	495	9.4	0.009
	No	1164	93.0	4786	90.6	
Coagulopathy	Yes	11	0.9	47	0.9	0.969
	No	1241	99.1	5234	99.1	
Antithrombotic medication	Yes	58	4.6	271	5.1	0.468
	No	1194	95.4	5010	94.9	
Anticoagulant medication	Yes	8	0.6	37	0.7	0.813
	No	1244	99.4	5244	99.3	
Liver cirrhosis	Yes	0	0	1	0.9	0.664
	No	20	100	106	99.1	
Anticoagulants	Yes	1	5.0	3	2.8	0.606
	No	19	95.0	104	97.2

**Table 3:** Unadjusted analysis of postoperative outcome comparing mesh versus non-mesh hiatoplasty before matching

		Mesh				
		Yes		No		
		*n*	%	*n*	%	*p*
Intraoperative complications—total	Yes	31	2.5	96	1.8	0.129
	No	1221	97.5	5185	98.2	
General complications—total	Yes	36	2.9	141	2.7	0.687
	No	1216	97.1	5140	97.3	
Postoperative complications—total	Yes	14	1.1	70	1.3	0.558
	No	1238	98.9	5211	98.7	
Complication-related reoperations	Yes	13	1.0	52	1.0	0.863
	No	1239	99.0	5229	99.0	
Recurrence on 1-year follow-up	Yes	68	5.4	303	5.7	0.674
	No	1184	94.6	4978	94.3	
Pain on exertion on 1-year follow-up	Yes	128	10.2	636	12.0	0.072
	No	1124	89.8	4645	88.0	
Pain at rest on 1-year follow-up	Yes	109	8.7	530	10.0	0.154
	No	1143	91.3	4751	90.0	
Pain requiring treatment on 1-year follow-up	Yes	93	7.4	469	8.9	0.099
	No	1159	92.6	4812	91.1	
Trocar hernia on 1-year follow-up	Yes	5	0.4	34	0.6	0.313
	No	1247	99.6	5247	99.4	
Secondary hemorrhage on 1-year follow-up	Yes	2	0.2	20	0.4	0.229
	No	1250	99.8	5261	99.6	
Blood effusion on 1-year follow-up	Yes	4	0.3	14	0.3	0.741
	No	1248	99.7	5267	99.7	
Infection on 1-year follow-up	Yes	14	1.1	60	1.1	0.957
	No	1238	98.9	5221	98.9	

### Covariable-adjusted matched pair analysis

**Fig. 2:**
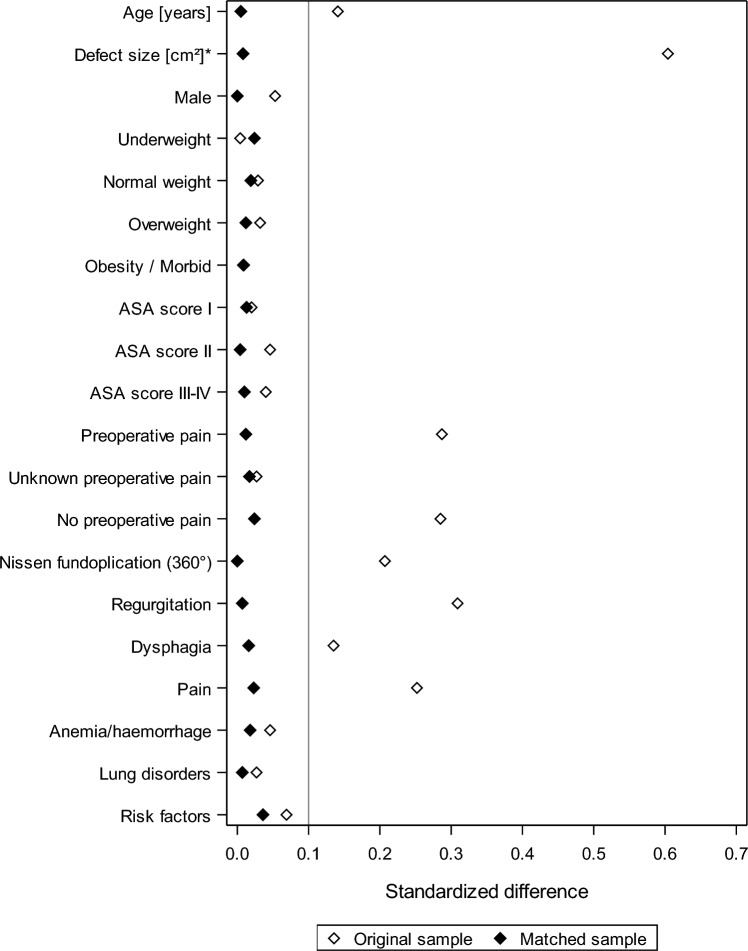
Standard differences between the matching variables both before (original sample) and after (matched sample) matching

Propensity-score matching was performed for 1252 with mesh to 5281 patients without mesh revealing 1207 matched pairs. Figure 2 shows the standardized differences between the matching variables both before (original sample) and after (matched sample) matching.

**Fig. 3:**
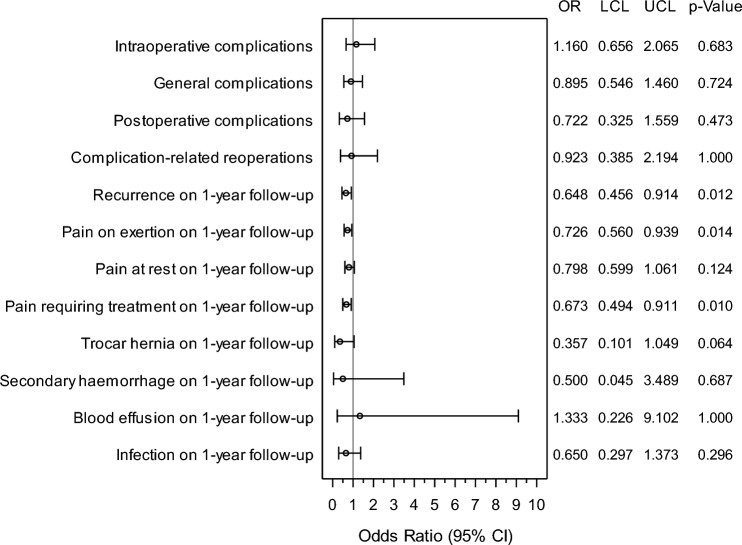
Forest plot—adjusted odds ratios (incl. confidence intervals) for all outcome parameter comparing mesh vs. non-mesh

That difference was well below 10% for all matching variables, attesting to a good balance between the groups for matched pair comparison for those variables. Table 4 demonstrates significant differences between mesh and non-mesh patients after matching. For recurrence there is a significant disadvantage for non-mesh patients. Here, 4.72% of mesh patients and 7.29% of non-mesh patients reported recurrence, when their matched patient reported no recurrence (discordant cases). Additionally in 0.33% both patients of a pair (concordant cases) had a recurrence. This corresponds to a significant deviation (*p* = 0.012). The same effect can be seen for pain on exertion (8.78% vs 12.10%; *p* = 0.014, with further 1.08% concordant cases) and pain requiring treatment (6.13% vs 9.11%; *p* = 0.010, with further 0.99% concordant cases). Regarding all other outcome parameters, no significant differences could be seen between mesh and non-mesh patients (Fig. 3).

**Table 4:** Outcome parameters between mesh and non-mesh patients after matching (*n* = 1.207 matched pairs)

No mesh		Mesh				*p*-Value
		Yes		No		
		*n*	%	*n*	%	
Intraoperative complications—total	Yes	1	0.08	25	2.07	
	No	29	2.40	1152	95.44	0.683
General complications—total	Yes	1	0.08	38	3.15	
	No	34	2.82	1134	93.95	0.724
Postoperative complications—total	Yes	0	0.00	18	1.49	
	No	13	1.08	1176	97.43	0.473
Complication-related reoperations	Yes	0	0.00	13	1.08	
	No	12	0.99	1182	97.93	1.000
Recurrence on 1-year follow-up	Yes	4	0.33	88	7.29	
	No	57	4.72	1058	87.66	0.012
Pain on exertion on 1-year follow-up	Yes	13	1.08	146	12.10	
	No	106	8.78	942	78.04	0.014
Pain at rest on 1-year follow-up	Yes	11	0.91	114	9.44	
	No	91	7.54	991	82.10	0.124
Pain requiring treatment on 1-year follow-up	Yes	12	0.99	110	9.11	
	No	74	6.13	1011	83.76	0.010
Trocar hernia on 1-year follow-up	Yes	0	0.00	14	1.16	
	No	5	0.41	1188	98.43	0.064
Secondary hemorrhage on 1-year follow-up	Yes	0	0.00	4	0.33	
	No	2	0.17	1201	99.50	0.688
Blood effusion on 1-year follow-up	Yes	0	0.00	3	0.25	
	No	4	0.33	1200	99.42	1.000
Infection on 1-year follow-up	Yes	0	0.00	20	1.66	
	No	13	1.08	1174	97.27	0.296

### Standardized differences for patients with and without follow-up-information

**Fig. 4:**
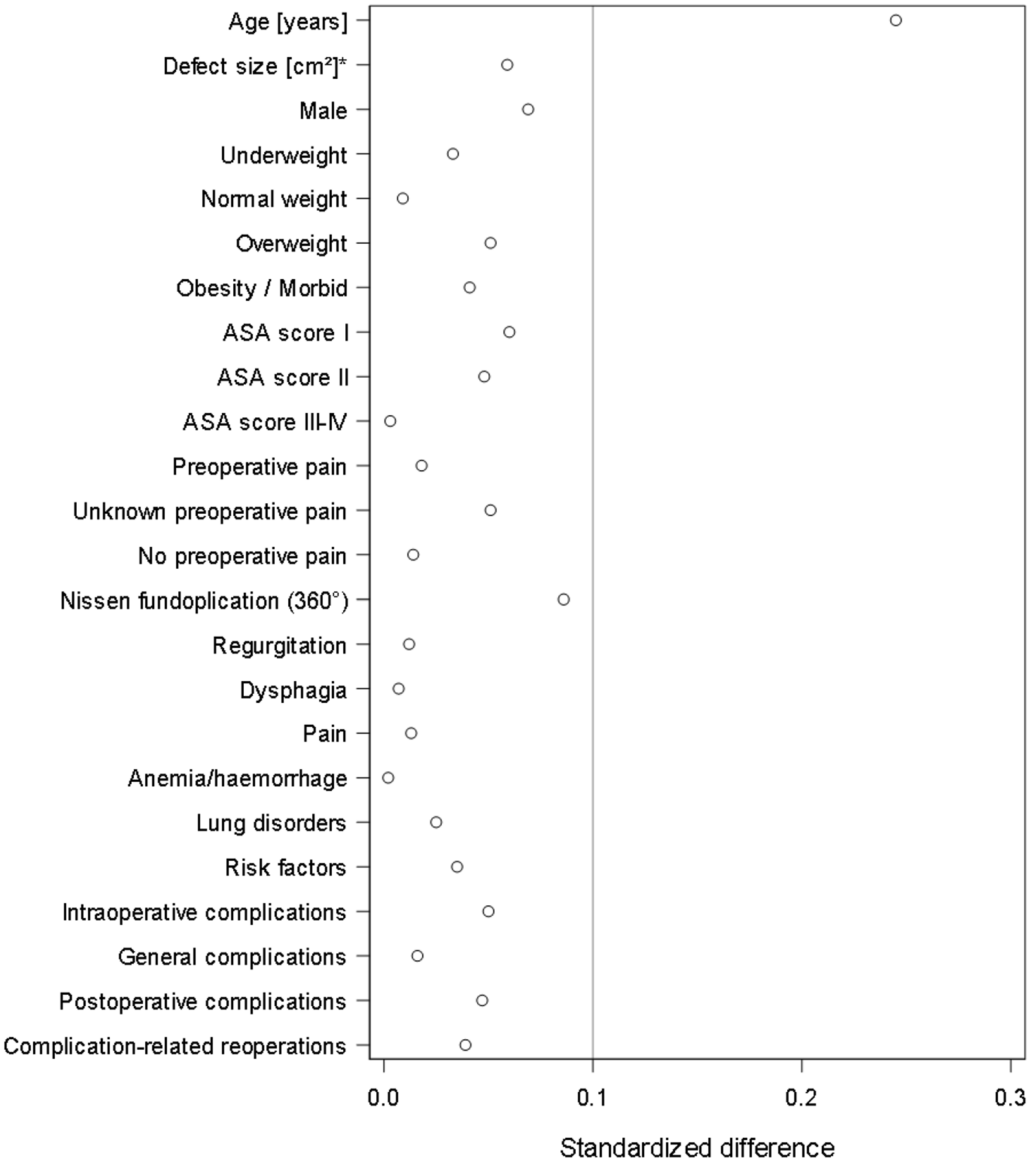
Scatter plot of standardized differences between patients with and without follow-up information

Figure 4 shows the results of the standardized differences for patients with (*n* = 6.533) and without (*n* = 3.368) follow-up information. Standardized differences above a value of 10% were found only for age. The patients in the analysis population were on average 3.5 years older. Standardized differences of less than 0.1 were found for all other variables, including the perioperative complication rates, and are thus not suggestive of any bias in patient selection.

### Defect sizes in the matched pair groups

After matching the defect sizes were in both groups with mean 15.9 cm^2^ comparable (Table 5).

**Table 5:** Presentation of mean values and ranges for age and defect size for mesh versus non-mesh hiatoplasty after matching

		Mesh		Stand. Diff	
		Yes	No	Matched sample	Original sample
Standardized differences					
Age [years]	Mean ± SD	57.9 ± 13.3	57.8 ± 13.3	0.005	0.141
Defect size [cm^2^]^a^	Mean [range of dispersion]	15.9 [14.1; 17.8]	15.9 [13.9; 17.8]	0.008	0.604

^a^ Logarithmic transformation: presentation of the back-transformed mean values and ranges (mean value ± SD)

### Types of meshes used in hiatoplasty

**Fig. 5:**
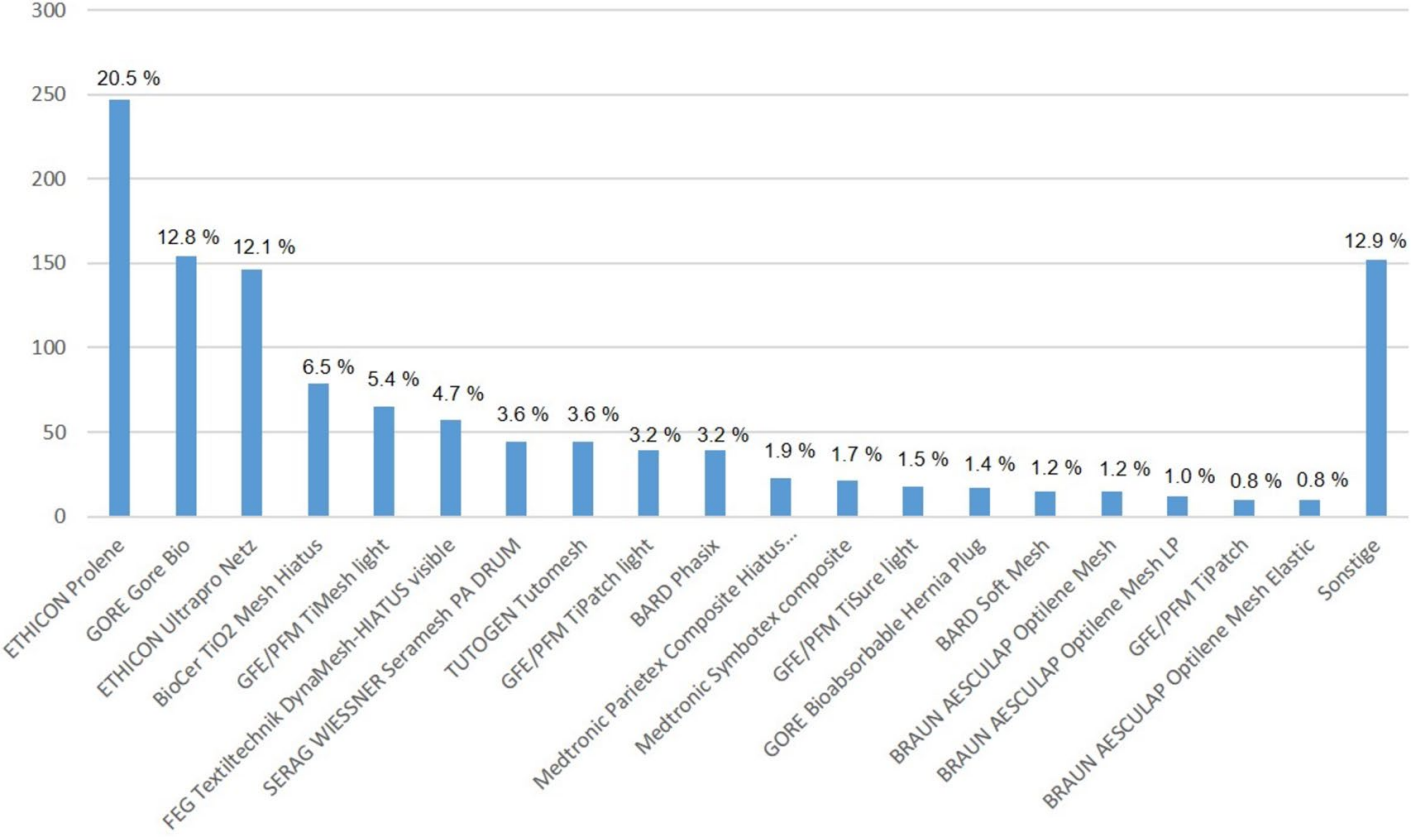
Distribution of the mesh types for mesh hiatoplasty after matching (*n* = 1.207 matched pairs)

A hughe variety of meshes has been used for augmentation of the hiatoplasty (Fig. 5). Beside polypropylene meshes, biological and biosynthetic meshes have been inserted for mesh hiatoplasty.

### Fixation of meshes

The majority of meshes for the augmentation for the hiatoplasty have been fixed with suture, tacks and glue (Table 6).

**Table 6:** Type of fixation in mesh hiatoplasty in 1.207 patients after matching

Fixation	*N*	%
No mesh fixation	46	3.81
Tacks + suture	57	4.72
Tacks + glue	3	0.25
Suture + glue	51	4.23
Tacks	393	32.56
Suture	403	33.39
Glue	254	21.04
Total	1207	100.00

### How was the recurrence diagnosed

In the subgroup of patients with non-mesh hiatoplasty *n* = 92 and in the subgroup of patients with mesh hiatoplasty *n* = 61 were diagnosed. The diagnosis was confirmed in *n* = 48 cases (31.4%) by radiology, in *n* = 106 cases (69.3%) by endoscopy and in *n* = 28 cases (18.3%) clinically.

## Discussion

This is the first large-scale study investigating mesh vs. non-mesh repair in laparoscopic type I hiatal hernia repair with GERD on the bases of data derived from a registry. We could demonstrate that in the subgroup of patients with large defect sizes mesh augmentation of the cruroplasty is associated with a significant lower recurrence rate at 1-year follow-up without increasing the risk of any complications.

Prevention of recurrence after hiatal hernia repair seems of utmost importance, since re-do operations are associated with an increased risk for complications [13]. In type I hiatal hernias there is consensus that sufficient anterior or posterior narrowing of the crura (cruroplasty) should be performed during every type I hiatal hernia repair [6, 14–16]. However, it remains controversial if the cruroplasty should be augmented with a mesh [10, 17–21], since the benefits of lower recurrence rates and long-lasting control of GERD symptoms should outweigh the risk of potential mesh-associated complications. In our study we could demonstrate, that using a mesh for augmenting the cruroplasty in the subgroup of patients with larger defect sizes is significantly associated with a reduced risk for recurrence. However, meshes and particularly synthetic meshes are suspect to increase the risk of erosion and fistula formation at the esophageus. Our results show up to 1-year follow-up that the risk for complications was comparable in the mesh and non-mesh group. This underlines the fact, the results from the studies suggesting a higher risk for complications in the mesh group must be interpreted with caution, mostly because only small case series have been published [6, 11, 12]. Furthermore, it must be considered that the mortality of a recurrence repair after failed type I hiatal hernia repair is sevenfold higher compared to the primary repair [22], highlighting the importance of a durable and stable primary repair. In a meta-analysis of RCTs comparing mesh augmentation of the cruroplasty vs. suture cruroplasty it has been shown that the risk for reoperation is significantly lower in the mesh group, while complication rates and operation time was comparably low in both groups [23]. Another meta-analysis revealed comparable results with lower recurrence rates after mesh augmentation of the cruroplasty with no increase in complications, questioning the omnipresent fear of mesh-associated complications [24]. In a risk–benefit-analysis of three RCTs and nine prospective controlled studies with 915 patients it could be demonstrated that the recurrence rate after mesh augmentation is almost halved in a follow-up period of 3 years with a decrease from 20.5% to 12.1% [25]. This reduction in recurrence rate corresponds to an absolute risk reduction of 8.4% or a number needed to treat (NNT) of 12 (95% CI 10.6–13.5). The reduction in recurrence rates appeared even more convincing in a subgroup analysis focusing on studies with a follow-up period of more than 2 years and the use of synthetic meshes. In this group, an life-long absolute risk reduction of 5.6% and an NNT of 18 (95% CI 13.3–27.3) was found with mesh augmentation of the cruroplasty [25]. The mesh-associated complications were rare (1.9%) and did not lead to a higher procedure-related complication rate or more overall complications of the procedure, which again contradicts arguments against the routine use of mesh augmentation in hiatal hernia surgery [25]. With our data we can only bring up arguments for the selective use of mesh augmentation of the cruroplasty in type I hiatal hernias with larger defects.

In our study population the risk of intra-, peri- and postoperative complications, secondary hemorrhage or blood effusion was not increased by using a synthetic mesh to reinforce the cruroplasty, which indicates that mesh-related complications seems to be not so frequent. In contrast, the non-mesh group with larger defect sizes had a significantly higher risk for recurrence, underlining the possible role of mesh augmentation in the prevention of a potential reoperation. Also, pain at rest and pain requiring treatment were significantly more frequent among the non-mesh group. Therewith, our findings may have some clinical implications. Pain following laparoscopic anti-reflux surgery occurs in over 20% of patients The preoperative consenting process in type I hiatal hernia patients specifically with larger defect sizes should include all arguments for and against mesh augmentation for the necessary cruroplasty. In younger patients, who may live long enough to experience a potential recurrence, a mesh augmentation of the cruroplasty should be discussed. In case the patients decline the use of mesh, the known risk of complications of a potential reoperation needs to be addressed and explained.

The large number of cases in registries are eminently suited to comparative studies since no patients are excluded due to strict criteria for clinical trial (high external validity) and, further potential influence factors on the outcome are also considered. However, since this is a registry-based study, there are some limitations.

There may also be some selection bias since choice of operative technique may depend on the surgeon’s expertise and different reimbursement for each surgical technique. Also, the health care systems of the countries recording their patients in the Herniamed Registry (e.g., Germany, Austria, Switzerland) may have specific limitations and biases regarding surgical treatment of hiatal hernias. However, in the Herniamed Registry these limitations are well-controlled and the following measurements are utilized to limit biases, limitations and incorrect or missing data, which could diminish generalizability of findings: signed contract with the responsible surgeon on each participating hospital for data correctness and completeness, display of missing data by the software, review of the perioperative outcome on 1-year follow-up and control of the data entry by experts as part of the certification process of hernia centers. The best safeguard is to match the data against other administrative data, literature or other registries [26]. Also, patient reported outcomes (PRO) as part of the follow-up process in the Herniamed registry are increasingly used [27, 28], adding value to the clinical evaluation and increasing patient satisfaction [29].

Since 1-year follow-up information is not available for one third of patients, it is necessary to rule out selection bias. On analysis of the standardized differences for patients with and without follow-up, values of 0.1 were routinely found, also for the perioperative complication rates, which argue against selection bias.

In conclusion, we could demonstrate that mesh augmentation of the cruroplasty during type I hiatal hernia repair with larger defect sizes is significantly associated with decreased rates of hernia recurrence, pain on exertion and pain requiring treatment.

## Data Availability

The data that support the findings of this study are available from the corresponding author (FK) upon reasonable request.
